# Generational differences in loneliness and its psychological and sociodemographic predictors: an exploratory and confirmatory machine learning study

**DOI:** 10.1017/S0033291719003933

**Published:** 2021-04

**Authors:** Drew Altschul, Matthew Iveson, Ian J. Deary

**Affiliations:** 1 Department of Psychology, The University of Edinburgh, Edinburgh, EH8 9JZ, UK; 2 Mental Health Data Science Scotland, Edinburgh, EH8 9JZ, UK; 3 Centre for Cognitive Ageing and Cognitive Epidemiology, University of Edinburgh, Edinburgh, EH8 9JZ, UK

**Keywords:** Aging, geriatric psychiatry, loneliness, machine learning, personality

## Abstract

**Background:**

Loneliness is a growing public health issue in the developed world. Among older adults, loneliness is a particular challenge, as the older segment of the population is growing and loneliness is comorbid with many mental as well as physical health issues. Comorbidity and common cause factors make identifying the antecedents of loneliness difficult, however, contemporary machine learning techniques are positioned to tackle this problem.

**Methods:**

This study analyzed four cohorts of older individuals, split into two age groups – 45–69 and 70–79 – to examine which common psychological and sociodemographic are associated with loneliness at different ages. Gradient boosted modeling, a machine learning technique, and regression models were used to identify and replicate associations with loneliness.

**Results:**

In all cohorts, higher emotional stability was associated with lower loneliness. In the older group, social circumstances such as living alone were also associated with higher loneliness. In the younger group, extraversion's association with lower loneliness was the only other confirmed relationship.

**Conclusions:**

Different individual and social factors might underlie loneliness differences in distinct age groups. Machine learning methods have the potential to unveil novel associations between psychological and social variables, particularly interactions, and mental health outcomes.

## Introduction

Loneliness increases on average as people age, making loneliness prevalent in older populations (Pinquart & Sorensen, 2001). Among older people, loneliness is particularly associated with early mortality risk (Holt-Lunstad, Smith, Baker, Harris, & Stephenson, 2015). Loneliness shows comorbidity with other conditions such as heart disease (Valtorta, Kanaan, Gilbody, Ronzi, & Hanratty, 2016), cognitive decline (Boss, Kang, & Branson, 2015), Alzheimer's disease (Wilson et al., 2007) and dementia (Zhou, Wang, & Fang, 2018).

On one hand, the putative cause of loneliness – social isolation – can be measured objectively to a degree by asking if an individual lives alone and/or by determining the characteristics of their social network (Holt-Lunstad et al., 2015). Loneliness, on the other hand, is a subjective, personal experience (de Jong Gierveld & Havens, 2004) that is assessed via one of several psychometric scales (Holt-Lunstad et al., 2015). People experience loneliness when they suffer from deficits in social contact when they experience a discrepancy between the quality and quantity of interpersonal relationships they would like to have, and the quality and quantity of the relationships they actually have (Pinquart & Sorensen, 2001). As people age, their social networks tend to shrink: they risk losing contact with partners and friends through death, they lose social roles, and they and their peers typically increase in frailty. All of this generally limits older persons' ability to maintain social contacts (Pinquart & Sorensen, 2001).

An increasing number of older people are living alone, and are more at risk of being lonely (Courtin & Knapp, 2017). Moreover, the average age of the world population is increasing (Lutz, Sanderson, & Scherbov, 2008) and as people live longer, distinct categories of older individuals will emerge. A common way to categorize people is as generations, such as the ‘Baby Boomers’ or ‘Silent Generation’. Different degrees of loneliness might be reported by different generations, and the factors predicting loneliness might not be the same across generations. For example, some evidence suggests that the risk factors for health conditions such as arthritis or obesity (Leveille, Wee, & Iezzoni, 2005) vary between the Boomers and Silents. Regarding loneliness, major social and cultural changes have occurred over the twentieth and twenty-first centuries, such as shrinking family sizes and a decline in multi-generation households (Victor et al., 2002). These are changes that could impact loneliness in mid-life and later. Therefore, the aim of this study was to examine which factors are related to loneliness in two distinct age groups of older adults.

Although common sense would predict a strong relationship, social isolation and loneliness are only weakly correlated (Hawkley, Burleson, Berntson, & Cacioppo, 2003). However, there is also evidence that other social factors such as low socioeconomic status (Pinquart & Sorensen, 2001), or psychological traits such as personality (Stokes, 1985), might have associations with loneliness. Another aim of the present study was to simultaneously analyze a number of plausible sociodemographic and psychological variables to find those strongly and robustly associated with loneliness. Moreover, loneliness is known to be synergistically affected by multiple factors, and to be associated with negative downstream health outcomes (Beller & Wagner, 2018). Synergistic effects, i.e. interactions, between variables are difficult to identify robustly (Gelman & Loken, 2016), and researcher degrees of freedom make objective variable selection yet more difficult to adhere to. For example, which variables and interactions to include, as well as what order to enter them into a regression model, are not clear-cut decisions, but can affect researchers' results. Machine learning provides techniques for selecting variables and quantifying their statistical importance that involve less human decision making. By analyzing a large set of variables across exploratory and confirmatory samples using state-of-the-art gradient boosting approaches, we searched for hitherto-unexplored interactions among variables associated with loneliness.

The current study followed three analytic steps. First, we used machine learning to identify the most statistically important psychological and social predictors that are associated with loneliness. By incorporating information from machine learning we could identify not only important single variables, but also interactions. Second, we sought replication of the associations with loneliness identified in our initial, exploratory samples in comparable independent, confirmatory samples. Third, we compared the predictive power of our machine learning models with models that were constructed using more conventional model-building approaches, thus evaluating the feasibility of identifying at-risk individuals. We did all of the above in two different age groups, i.e. with two exploratory samples and two confirmatory, replication samples.

## Methods

### Cohort selection

We identified four cohorts with data on loneliness and a large set of comparable psychological and sociodemographic variables that included, and were relevant to, loneliness. We wanted our analyses to have access to the same set of variables in each sample, thereby limiting researcher degrees of freedom in the selection of variables. Two longitudinal cohorts were used for exploration (also referred to as training datasets), and two others were used for replication (also referred to as confirmatory or test datasets). The results of exploratory analyses in the first older (age 70–79 years) cohort were confirmed in a second older-age cohort. The results of exploratory analyses in a younger, mid-life (aged between 45 and 69) cohort, were confirmed in a second cohort with the same age range.

A goal was to select cohorts that surveyed at least 500 individuals, a quantity both sufficiently large and practical. The larger, higher-powered cohorts of each pair were used for confirmation to protect against effect size deflation that often occurs when attempting to replicate effects from one sample to another.

### Older samples

#### Exploratory older sample: Thirty-Six Day Sample

The Thirty-Six Day Sample (36DS) is a representative subsample of children born in Scotland in 1936, consisting of individuals selected according to their dates of birth being on one of the first 3 days of each month (i.e. 36 days throughout the year). The original sample size was 7277, and in 2012 and 2013, members of 36DS were traced through the UK National Health Service Central Register and contacted by letter (Brett & Deary, 2014). A total of 722 individuals completed a detailed questionnaire and 365 individuals later completed a telephone interview (Brett & Deary, 2014; Deary & Brett, 2015) at mean age 77.5 (median 77.7).

#### Confirmatory older sample: Lothian Birth Cohort of 1936

The Lothian Birth Cohort of 1936 (LBC1936) has origins in common with 36DS. Potential participants were contacted by letter between 2004 and 2007, and1091 individuals born in Scotland in 1936 were recruited to Wave 1 of the LBC1936 study, at a mean age of 70 years (Taylor, Pattie, & Deary, 2018). Other cognitive, psychosocial, lifestyle, and other data were collected at wave 1 and in three subsequent waves, at ages 73, 76, and 79. In total 76 members of LBC1936 were also part of 36DS; these individuals were removed from LBC1936 and thus only used in exploratory analyses.

### Younger samples

#### Exploratory younger sample: Healthy Ageing in Scotland

Healthy Ageing in Scotland (HAGIS) is a proposed longitudinal study of older individuals living in Scotland, currently consisting of a pilot sample of 1000. Participants were identified by household; the household was contacted by letter and everyone living there was invited who met one of the following two conditions: (1) adults who were at least 50 years of age at the time of data collection, (2) partners of adults aged 50 years or older who were themselves 45 years or older (Douglas, Rutherford, & Bell, 2018). Despite including individuals older than 70, to ensure that there was no age overlap between the younger and older samples after variables were selected and processed, individuals older than 69 years were removed from our analytic HAGIS sample, leaving 612 participants. Exact ages were not available in HAGIS, though enough information on bands was provided to remove individuals older than 69.

#### Confirmatory younger sample: English Longitudinal Study of Ageing

The English Longitudinal Study of Ageing (ELSA) is a panel study of a representative cohort of English men and women (Steptoe, Breeze, Banks, & Nazroo, 2012). Started in 2002, the initial sample was recruited from the list of participants in the Health Survey for England. Individuals were contacted and invited if they would be 50 years or older by the start of the first wave of data collection. We analyzed wave 2 of ELSA, the first with a measure of loneliness, which included 9432 participants. These data were collected in 2004 and 2005 when participants were 59 years old on average. As with HAGIS, for analysis, individuals older than 69 at the time of wave 2 data collection were removed from our analytic ELSA sample, leaving 6106 participants with mean and median ages of 59.3 and 59. The samples are described in more detail in the online Supplementary Materials.

### Loneliness

Different loneliness assessments were available in different samples. In the older samples (36DS and LBC1936), a single question was asked. In 36DS, participants read, ‘Loneliness can be a serious problem for some people and not for others. At the present moment do you feel lonely?’ and endorsed one of ‘Never’, ‘Seldom’, ‘Only occasionally’, ‘Quite often’ or ‘Most of the time’. LBC1936 participants were asked ‘At the present moment do you feel lonely?’ and endorsed one of ‘Never’, ‘Seldom’, ‘Only occasionally’, ‘Quite often’ or ‘Most of the time’. Responses in both cohorts were ordinal assigned values of 1 to 5 with, ‘Most of the time’ registering as a 5.

HAGIS used a six-item loneliness scale (de Jong Gierveld & Havens, 2004) that was composed of the following items, all rated ‘No’, ‘More or less’ or ‘Yes’: ‘I experience a general sense of emptiness’, ‘I miss having people around me’, ‘I often feel rejected’, ‘There are plenty of people I can rely on when I have problems’, ‘There are many people I can trust completely’, and ‘There are enough people I feel close to’. These items were averaged to generate an overall loneliness score between 1 and 3, with intervening values. ELSA used three items from the University of California, Los Angeles Modified Loneliness Scale: ‘I feel left out’, ‘I feel isolated’, and ‘I lack companionship’ (Shankar, Hamer, McMunn, & Steptoe, 2013). Also rated on a three-point scale – ‘Hardly ever or never’, ‘Some of the time’, and ‘Often’ – these items were averaged to create a comparable 1–3 loneliness scale.

### Personality

36DS, LBC1936, and HAGIS all used versions of the International Personality Item Pool (IPIP). The IPIP labels for the big five personality dimensions are Emotional Stability, also known as Neuroticism, Extraversion, Agreeableness, Conscientiousness, and Intellect, also known as Openness or Openness to Experience. 36DS used the 20-item mini-IPIP (Donnellan, Oswald, Baird, & Lucas, 2006). LBC1936 and HAGIS used the 50-item IPIP (Goldberg et al., 2006; Gow, Whiteman, Pattie, & Deary, 2005). Scoring systems for personality in each sample are fully described in the online Supplementary materials.

ELSA used a version of the Midlife Development Inventory; this was originally designed for the use in the Midlife in the US study and used in this format in the Health and Retirement Study (Lachman & Weaver, 1997). Personality in ELSA was taken at wave 5, approximately 6 years after wave 2, which was used for most of our baseline variables from ELSA. The big five factors of personality have been shown to be stable over this time interval (Costa & McCrae, 1988) and where personality differences do change, the change is modest (Roberts, Walton, & Viechtbauer, 2006).

### General cognitive function

General cognitive function was assessed using several widely used tests in each cohort. The particular tests that were available differed in each cohort (Douglas et al., 2018; Shankar et al., 2013; Taylor et al., 2018). In each cohort, we conducted a principal component analysis and took the first component of all test scores and used that as our measure of cognitive function in subsequent analyses. In 36DS all tests scores weighted above 0.61 on the first component; the highest was Word Recall, weighted at 0.79. The variance explained by the first component was 45%. In LBC1936, the lowest weighting was for Inspection Time (0.40), and the highest was shared by the NART and WTAR (0.73). The variance explained by the first component was 38%. In HAGIS, weightings ranged from 0.52, for Vocabulary, to 0.76, for Word Recall. The variance explained by the first component was 44%. In ELSA, the highest weighting was for Letter Cancellation (number correct) at 0.82, and 0.33 for Letter Cancellation (number missed). The total variance explained by the first component was 52%. A full description of the cognitive test variables used in each sample and the resultant principal component analyses is available in the online Supplementary materials.

### Subjective health

In each cohort, subjective health was assessed with a single question asking the respondent to rate their health on a 5 point scale (Nummela, Seppänen, & Uutela, 2011). In LBC1936, a similar item from the World Health Organization Quality of Life questionnaire was used. Poorest health was represented by 1 and the best health was represented by 5.

### Sociodemographic variables

Different social class variables were available depending on the sample. 36DS and LBC1936 used the 6 ordinal groups of the Standard Occupational Classification (SOC) 2000 (Elias, McKnight, & Kinshott, 1999). ELSA used the National Statistics Socioeconomic Classification (NS-SEC), which classifies occupations on an eight-point ordinal scale (Banks, Karlsen, & Oldfield, 2003). Occupational social class was not available in HAGIS, so we used the complete scale of the Scottish Index of Multiple Deprivation (SIMD) revised in 2016, instead.

In the older generation samples, education was measured as the number of full-time years of education that a participant had completed. In the younger generation samples, only variables reflecting degree of education through qualifications were available, though they were broadly comparable between the Scottish and English samples used. The Scottish Vocational Qualifications used in HAGIS and National Vocational Qualifications used in ELSA were compared (https://eal.org.uk/support/document-library/7-uk-qualifications-comparison-table/file) and arranged on a common ordinal scale.

Number of children was readily available as a single variable in all samples, as was sex. Whether a participant was married, partnered, divorced, single, etc., was coded categorically in the datasets and converted to binary dummy variables for analysis (being married or partnered was the reference category). Similarly, who, if anyone, a participant was living with was also coded in categorical variables, and converted to binary dummy codes (living with a spouse or partner was the reference category). More details on variables cleaning and processing are available in the supplemental materials.

### Exploratory analyses

We wanted our models to select variables with minimal user input or bias, and so used extreme gradient boosted modelling (XGBM) to identify which variables were related to loneliness in our exploratory samples. XGBM is a machine learning technique that sequentially applies a decision tree fitting algorithm to training data that it then reweights across iterations. The final result of the algorithm is a weighted majority of the sequence of trees that are produced (Chen, He, Benesty, Khotilovich, & Tang, 2015). Across all trees in an XGBM, individual variable importance can be assessed. Because multiple branches in constituent trees are comparable to interactions, XGBMs can also identify important interactions between variables.

We used ‘caret’ (Kuhn 2008) to train separate XGBMs for the older and younger generations, with a wide range of parameter values to identify the best model, based on the mean squared error. See the supplemental data for complete XGBM output including the importance of each variable and interaction included in the best-fit XGBMs. The most important variables of the XGBM were sequentially added to ordinal regression models (ORMs) of the exploratory data. In each cohort, the highest importance individual variables were added first, until adding a variable no longer improved the ORM fit. Fit improvement was assessed via likelihood ratio tests, comparing the χ^2^ values of the sequential, nested models. Once we had done this for individual variables, we followed the same process for interactions. If our XGBMs suggested the inclusion of an interaction involving an individual variable that was not already included in the ORM, the individual variable(s) was also added to the ORM for ease of interpretation.

### Confirmatory analyses

We cross-validated our exploratory analyses for each of the older and younger cohort pairs. The final models in each generation's exploratory cohort were fitted in the confirmatory datasets, and effect sizes, standard errors, test statistics, and confidence intervals were compared between the exploratory and confirmatory models. These models were the ‘in-sample ORMs’ described below.

We also wished to make predictions from our models, and so calculated prediction scores (mean squared error) for loneliness in four different models each for both generations' confirmatory cohorts. Every model can predict its outcome (in this study, loneliness) values for every participant, and the ‘error’ of these predictions is the difference between predicted and real values. Mean squared error is thus an overall score for the predictive power of an individual model; the best models are those than minimize mean squared error. Mean squared error is also known as a ‘loss function’, which means that it represents how ‘wrong’ an estimated outcome actually is.

The first models were null, intercept only, ORMs. The second and third were the XGBMs and ORMs, both fitted with the exploratory data. These XGBMs and ORMs are referred to as ‘out-of-sample’ models because they use parameters determined by the exploratory datasets (the ‘out’ samples), but as with all of our confirmatory models, they predict outcome values using the confirmatory datasets (the ‘in’ samples). The fourth pair of models were ORMs with the exact same predictors as the third pair of models. The salient difference was that these ORMs were fitted with the confirmatory data and predicted those same confirmatory outcome data, making these ‘in-sample’ models.

## Results

### Demographic characteristics

**Fig. 1.**
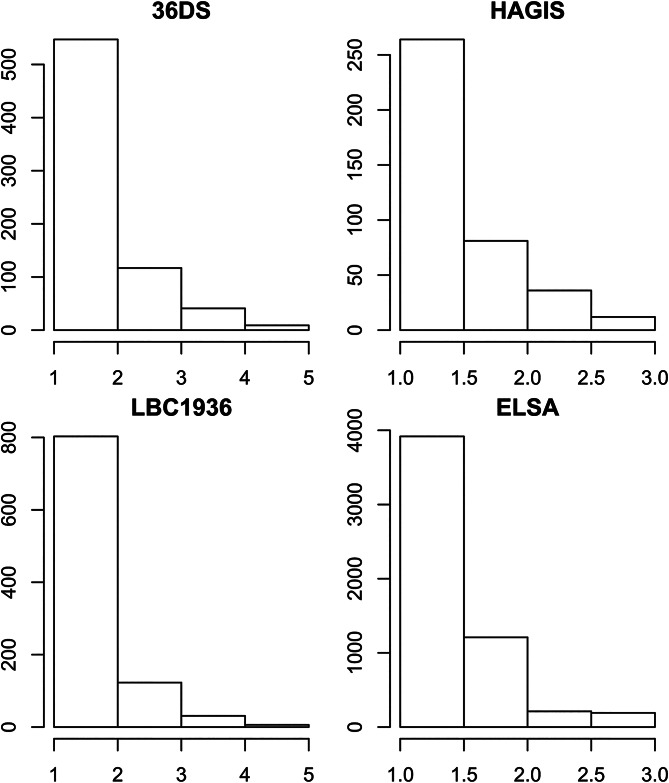
Histogram of loneliness across all four analytic samples. The *y*-axes differ between the older and younger samples because loneliness was measured using a different number of items between age groups, though the same items were used within age groups. 36DS, Thirty-six Day Sample; LBC1936, Lothian Birth Cohort of 1936; HAGIS, Healthy Ageing in Scotland; ELSA, English Longitudinal Study of Ageing.

Descriptive statistics for all variables in all four cohorts are given in Table 1. Loneliness was similar within and between generations. Within generations, means and s.d. for loneliness were comparable, although in both confirmatory samples the means and s.d. were lower than in the smaller exploratory samples. Different generations used different loneliness scales, so to compare them, we plotted histograms for all four samples with the same number of bins (Fig. 1). The overall distributions were similar in all four samples, although there appeared to be more high loneliness individuals in the younger samples than the older sample.

**Table 1.** Descriptive statistics for four samples and all overlapping variables of interest.

				Older								Younger				
	Exploratory – 36DS			Confirmatory – LBC1936					Exploratory – HAGIS			Confirmatory – ELSA				
	*N* = 792			*N* = 1015					*N* = 612			*N* = 6106				
Predictor	*n*	mean	s.d.	*n*	mean	s.d.	*t*	*p*	*n*	mean	s.d.	*n*	mean	s.d.	*t*	*p*
Loneliness	714	1.85	0.98	893	1.68	0.87	3.65	<0.001	393	1.48	0.47	5530	1.35	0.49	0.21	0.831
Extraversion	707	11.65	3.50	884	10.65	3.52	5.69	<0.001	393	3.09	0.72	4122	3.96	0.68	−21.99	<0.001
Agreeableness	708	16.17	2.47	882	15.54	2.71	4.88	<0.001	394	3.83	0.49	4121	4.39	0.60	−20.87	<0.001
Conscientiousness	708	15.75	2.71	884	14.14	2.99	11.29	<0.001	394	3.72	0.64	4121	4.13	0.60	−12.16	<0.001
Emotional Stability	707	12.93	3.31	881	12.32	3.85	3.42	<0.001	394	3.29	0.76	4117	3.37	0.74	−1.88	0.061
Intellect	706	13.39	2.70	880	11.94	2.84	10.33	<0.001	394	3.33	0.57	4119	3,60	0.68	−8.93	<0.001
Cognitive function	348	0	1.00	934	0.03	0.99	−0.42	0.670	545	0.35	0.89	6106	0.25	0.89	2.33	0.020
Subjective health	718	3.21	0.99	892	3.71	0.94	−10.42	<0.001	611	3.05	1.15	6040	3.31	1.12	−5.21	<0.001
Social class	615	2.49	0.88	996	2.41	0.92	1.66	0.096	612	3545	1874	6047	3461	1963	1.045	0.296
Education	747	11.26	2.34	1015	10.74	1.12	5.54	<0.001	594	4.70	2.03	2953	4.37	1.87	3.70	<0.001
Number of children	713	2.32	1.26	896	2.15	1.27	2.75	<0.001	386	1.76	1.24	4491	2.17	1.44	−6.07	<0.001
	*n*	cases		*n*	cases		χ^2^	*p*	*n*	cases		*n*	cases		χ^2^	*p*
Sex (male)	792	412		1015	515		0.24	0.622	610	267		6106	2692		0.01	0.914
Married or partnered	720	457		1014	741		17.74	<0.001	611	419		6106	4520		8.20	0.004
Single	720	34		1014	62		1.31	0.253	611	66		6106	334		27.25	<0.001
Widowed	720	188		1014	135		44.65	<0.001	611	39		6106	423		0.17	0.672
Divorced or separated	720	41		1014	76		1.89	0.169	611	87		6106	829		0.15	0.694
Living with spouse/partner	718	465		892	662		16.48	<0.001	492	311		6106	4829		65.74	<0.001
Living alone	718	231		892	208		15.28	<0.001	492	153		6106	999		67.59	<0.001
Living with family*	718	18		892	22		0.00	1	492	25		6106	268		0.36	0.546
Living with others	718	4		-	-				492	3		6106	10		2.62	0.106

36DS, Thirty-six Day Sample; LBC1936, Lothian Birth Cohort of 1936; HAGIS, Healthy Ageing in Scotland; ELSA, English Longitudinal Study of Ageing.

All variables are on the same scale within generations, but not necessarily between. In the older generation, loneliness is on a scale from 1 to 5, but from 1 to 3 – with many intervening values – in the younger generation. Personality dimensions are on a scale from 4 to 20 in older participants; from 1 to 5 in younger participants. Social class runs from 1 to 5 in the older participants, but in HAGIS, the variable is SIMD16 household score. In ELSA, social class was originally 1 to 8, but scaled to match the SIMD16.

*36DS participants ‘living with family’ also includes ‘living with child’.

The exploratory samples had about the same proportion living alone (32% in 36DS and 31% in HAGIS, χ^2^ = 0.11, *p* = 0.740), while the proportions were lower in the confirmatory samples (23% in LBC1936 and 16% in ELSA, χ^2^ = 25.91, *p* < 0.001) (Table 1). These figures can be contrasted with those people in the samples who are not married or partnered. Again, the exploratory cohorts are similar in this respect (36% in 36DS and 31% in HAGIS are not married or partnered, χ^2^ = 3.60, *p* = 0.058), as are the confirmatory cohorts (27% in LBC1936 and 26% in ELSA, χ^2^ = 0.36, *p* = 0.550). However, in the younger cohorts, the number of divorced individuals is nearly double the number of widowed individuals, whereas in the older cohorts the opposite is true, and the number of widows and widowers far exceeds the divorcees.

### Models of factors associated with loneliness in the older generation

**Fig. 2.**
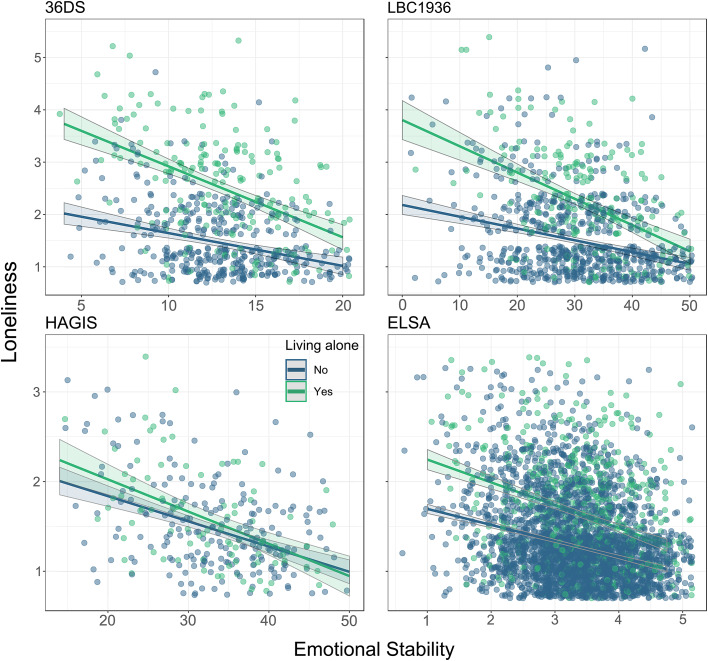
Emotional stability *v*. loneliness scores, stratified by whether one lives alone, plotted in all four cohorts. Emotional stability is presented on the scale the data were collected at in each sample, which differs due to the Likert scaling and number of items used: Emotional stability in 36DS ranges from 4 to 20, in LBC1936 ranges from 0 to 50, in HAGIS ranges from 10 to 50, and in ELSA ranges from 1 to 5. 36DS, Thirty-six Day Sample; LBC1936, Lothian Birth Cohort of 1936; HAGIS, Healthy Ageing in Scotland; ELSA, English Longitudinal Study of Ageing.

Among the older generation, our models of loneliness – XGBMs and subsequent ORMs – identified several main effects and two interactions in 36DS (Table 2). Loneliness was negatively associated with emotional stability, negatively with subjective health, positively with whether a participant was widowed, and positively with whether a participant was living alone. Both emotional stability and sex independently interacted with living alone: less emotionally stable individuals who lived alone were relatively lonelier and men who lived alone were also lonelier. We confirmed three main effects: lower emotional stability, lower subjective health, and living alone, which were all associated with higher loneliness. The two interactions were also confirmed. While anyone living alone tended to be lonelier, women who were not living alone were more likely to be lonely than men not living alone, and men who were living alone were more likely to be lonely than women living alone (online Supplementary Fig. S1). The other interaction effect was with emotional stability: less emotionally stable people who lived alone were lonelier than more emotionally stable participants who lived alone (Fig. 2, upper two panels); at the highest levels of emotional stability, low levels of loneliness were reported, whether the person was living alone or not. The exploratory ORM explained 41.2% of the variation in loneliness, and the confirmatory ORM explained 27.7%.

**Table 2.** Ordinal regression analyses of loneliness in the exploratory and confirmatory samples, in older and younger generations

Older generation										
	Exploratory – 36DS					Confirmatory – LBC1936				
Predictor	*B*	s.e.	*t*	*β*	95% CI	*B*	s.e.	*t*	*β*	95% CI
Emotional stability	−0.143	0.035	−4.137	−1.279	(−1.649 to −0.917)	**−0.140**	**0.023**	**−6.122**	**−1.255**	**(−1.565 to −0.950)**
Subjective health	−0.343	0.091	−3.752	−0.665	(−1.014 to −0.319)	**−0.350**	**0.079**	**−4.434**	**−0.656**	**(−0.947 to −0.366)**
Social class	0.169	0.096	1.751	0.300	(−0.035 to 0.636)	−0.072	0.079	−0.913	−0.131	(−0.413 to 0.150)
Sex (male)	−0.647	0.211	−3.071	−0.260	−0.597 to 0.075)	−0.120	0.165	−0.714	0.050	(−0.237 to 0.337)
Widowed	0.592	0.276	2.147	0.509	(0.044 to 0.976)	0.410	0.254	1.611	0.280	(−0.061 to 0.621)
Living alone	3.562	0.751	4.740	2.025	(1.533 to 2.527)	**2.676**	**0.612**	**4.374**	**1.506**	**(1.156 to 1.859)**
Living alone × ES	−0.157	0.055	−2.857	−0.969	(−1.639 to −0.307)	**−0.102**	**0.045**	**−2.266**	**−0.656**	**(−1.228 to −0.092)**
Living alone × Sex	1.235	0.364	3.388	1.145	(0.484 to 1.810)	**0.736**	**0.334**	**2.199**	**0.620**	**(0.067 to 1.174)**
Residual deviance	1159.931					1635.17				
AIC	1182.931					1659.17				
pseudo *R*^2^	0.412					0.277				
Younger generation										
	Exploratory – HAGIS					Confirmatory – ELSA				
Predictor	*B*	s.e.	*t*	*β*	95% CI	*B*	s.e.	*t*	*β*	95% CI
Emotional Stability	−1.011	0.130	−7.78	−1.540	(−1.932 to −1.156)	**−0.683**	**0.046**	**−14.91**	**−0.797**	**(−1.134 to −0.870)**
Extraversion	−0.753	0.129	−5.81	−1.133	(−1.519 to −0.754)	**−0.590**	**0.048**	**−12.34**	**−0.924**	**(−0.924 to −0.670)**
Residual Deviance	1610.181					9916.629				
AIC	1646.181					9936.629				
pseudo *R*^2^	0.265					0.125				

36DS, Thirty-six Day Sample; LBC1936, Lothian Birth Cohort of 1936; HAGIS, Healthy Ageing in Scotland; ELSA, English Longitudinal Study of Ageing; ES, Emotional Stability.

Pseudo *R*^2^s are Nagelkerke adjusted. *β*s are standardized betas. Bolding indicates significant predictors, only in the confirmatory samples. Predictors in the exploratory samples were a subset of those used in the machine learning training, based on the relative statistical importance of each variable in the machine learning models, and included in these parametric models if they improved the χ^2^ of the model.

### Younger generation

Across HAGIS and ELSA, we identified two main effects and confirmed both of them (Table 2). As in the older generation, there was a main effect of emotional stability such that less emotionally stable people were more likely to be lonely (Fig. 2). Moreover, an additional personality dimension was associated with loneliness: participants with higher extraversion were less likely to be lonely. There was no significant emotional stability by living alone interaction, by contrast with the older samples. The exploratory ORM explained 26.5% of the variation in loneliness, and the confirmatory ORM explained 12.5%. These models explain less of the variance in loneliness than the corresponding models used for the older cohorts, probably because of how many fewer predictors were included in the ORMs – 2 in the younger cohorts *v.* 8 in the older cohorts.

### Predictive scoring

Within each generation, the predictive power of the generated models was examined by contrasting the mean squared error for loneliness (Table 3). In particular, null (intercept-only) ORMs were contrasted with the final out-of-sample XGBMs fitted with exploratory data, final out-of-sample ORMs fitted with exploratory data, and final in-sample ORMs fitted with confirmatory data.

**Table 3.** Cross-validation scores for models predicting loneliness in the confirmatory samples

		Out-of-sample		In-sample
	Null	Boosted model	Ordinal model	Ordinal model
Older generation	1.172	0.563	0.709	0.713
Younger generation	0.915	0.857	0.772	0.851

All numbers are mean squared error scores. The older generation confirmatory sample is the Lothian Birth Cohort of 1936, and the younger generation confirmatory sample is the English Longitudinal Study of Ageing. The null models are intercept only models. Out-of-sample models were fitted to the exploratory samples and their parameters were determined by those samples. The In-sample models were fitted to the confirmatory samples and thus the models' parameters and prediction scores were determined by the same data.

In the older generation, our null ORMs – representing the effectiveness of fitting only an intercept – were not very good at predicting loneliness compared to the more informed models. The XGBM performed better than the out-of-sample ORM: MSE = 0.563 *v.* 0.709, indicating that the better performing XGBM would, on average, over or underestimate loneliness by about ¾ of a point on the loneliness scale. We stopped adding to the ORM when we no longer improved fit, which suggests that the many smaller main effects and interactions used in the XGBM were of real predictive utility, even though they were not strong enough to be included in our ORM.

This was not the case in the younger generation: the best performing model was the out-of-sample ORM (MSE = 0.709, indicating the model was usually off by about 0.85 points on the loneliness scale), but the differences in performance between all the younger generation models, including the null model, was much less. In the case of the XGBM, this is understandable: when tuning the XGBM, we determined that the best parameters for training did not include interactions, only main effects. This makes for a much simpler boosted model, and so one closer in predictive power to the null ORM. Nevertheless, in the younger generation it appears that there are fewer variables that predict loneliness (Table 2), compared to the models in the older generation.

## Discussion

Our analyses showed that a range of psychological and social factors are associated with loneliness in later life. The best predictive models in each generation (45–69 years old, and 70–79 years old) achieved good prediction scores, similar to the best mean squared errors achieved in comparable models of sociodemographic and psychological variables predicting personality traits (Altschul, 2019). In-sample explained variation (*R*^2^-type measures) and out-of-sample prediction scores (MSE) are complementary metrics for evaluating the strength of a model. In both generations' models, pseudo-*R*^2^s and MSEs demonstrated that the models have real explanatory power, although the metrics for the models of the younger generation were not as good. This suggests that loneliness in 45–69 year-olds is harder to predict.

Despite this, loneliness was similarly prevalent in the younger and older generations. The underlying sources of difference in predictive power between generations may be in the associated variables: apart from emotional stability, the factors associated with loneliness in our best fit models were different. Higher emotional stability, otherwise known as neuroticism (in reverse), was associated with less loneliness. Emotional stability was the only variable whose influence spanned both generations, and also had one of the stronger effect sizes. Higher emotional stability has been repeatedly shown to be associated with less loneliness (Hawkley et al., 2003; Stokes, 1985). This association likely stems from a common endogenous source of negative affect (Amichai-Hamburger & Ben-Artzi, 2003; Stokes, 1985), with some genetic contribution (Abdellaoui et al., 2019). Similarly, subjective health reports are also associated with loneliness (Sundström, Fransson, Malmberg, & Davey, 2009) as well as negative affect (Watson & Pennebaker, 1989), so the association between lower subjective health and higher loneliness in the older generation may be in part due to higher negative affect.

Living alone, a variable tapping into social isolation, was associated with more loneliness in the older generation, with the largest effect sizes of any variable. Moreover, the difficultly in living alone could be eased or exacerbated by two other key variables: sex and emotional stability. Less emotionally stable individuals appeared to be less able to cope with living alone, and men living alone were more at risk for being lonely. In the older group, many more individuals living alone were widowed, as opposed to divorced. Coping with the grief and stress of losing one's partner or spouse is challenging, and more emotionally stable tend to also be more resilient grievers (Mancini, Sinan, & Bonanno, 2015). Emotionally stable people tend to experience fewer negative emotions (Larsen & Ketelaar, 1991), and daily experience of less negative emotion and more positive emotion aids resilient individuals in their ability to recover from major stressors (Ong, Bergeman, & Boker, 2009).

Among the younger generation, no variables indicating social circumstances or isolation were significantly associated with loneliness. These variables may not be important in the younger group because they have not had the same opportunities or exposures as the older samples. On the other hand, higher extraversion, a personality variable indicating social network strength, was associated with lower loneliness, as was higher emotional stability. Lower extraversion has been shown to be associated with loneliness through the mediation of social network variables (Stokes, 1985), but despite giving our models access to many social variables including family size and social class, extraversion was more important.

The difference between the impacts of social circumstances *v.* socially relevant personality traits suggests a generational difference in how older individuals are affected by loneliness. There are at least two possible non-mutually exclusive explanations for this. First, as individuals age, though they may be extraverted they may not be physically able to visit or entertain others. One's partner might become a more important cornerstone for social connection, and an individual's particular social personality profile might become less important; that is, the social environment might not afford the expression of personality differences so much at the older ages. Second, individuals in the younger generation, under 69 years-old, may have different social and cultural needs. Rather than relying on their family as much, social contact might be expected to come more from their wider social network, and individuals who are less extraverted will have fewer social contacts, and therefore be lonelier. A notable potential source for extraverted individuals to find wider social connections is the internet (Amichai-Hamburger & Ben-Artzi, 2003).

The present study has some limitations. All four samples were from the UK, and three were from Scotland, which may limit the generalizability of our study. Nevertheless, the findings are all in line with those of other studies from a variety of different countries (Amichai-Hamburger & Ben-Artzi, 2003; Hawkley et al., 2003; Pinquart & Sorensen, 2001; Stokes, 1985). Although our samples all included hundreds of participants, the effective sample size for some models was limited by a lack of participation in some aspects of the surveys. This may have limited our ability to identify some of the weaker main effects or interactions. It was also not possible to formally compare models between samples from different generations, though our variables measured the same things, there were critical differences in some scales, particularly the outcome measure, loneliness. The loneliness outcome was measured using only a single item for the older samples, and while it was composed of the same three questions in the younger sample, the full measures for loneliness differed in HAGIS and ELSA. Moreover, even when using the same scales there can be differences: both confirmatory samples had lower average loneliness than in their respective exploratory samples. We expect that a certain amount of noise from using different questionnaires is included in our results; while all comparable measures assessed the same underlying constructs, many of our variables were not all collected the same way (e.g. personality, education).

Although the two pairs of cohorts were matched in terms of their age ranges, even in terms of their age characteristics they are not copies of each other. The two older cohorts are drawn from the same subpopulation: all 11-year-old children who were in school in Scotland on 4 June 1947. However, the samples were not followed up at the same times, so the average age of a 36DS participant was 77.5 and the average age of a LBC1936 participant was 70. This is reflected in some sample differences, e.g. age is associated with loneliness, so the average loneliness in 36DS is slightly higher than LBC1936. On the other hand, detailed ages were not available in HAGIS, so while we know the range, we cannot compare HAGIS’ age characteristics to ELSA's, or those of the other samples.

The exploratory–confirmatory approach is also not immune to overlooking notable variables in the confirmatory samples. In confirmatory samples we are usually unaware of what we may be missing, but, in this case, visualization helps. For example: in Fig. 2, although we saw no significant association between living alone and loneliness in HAGIS, it appears there may be such an association in ELSA. Yet, to follow our pre-determined strict guidelines for analysis, it is beyond the scope of this paper to confirm this analytically. We thus note that, even within generations, sample characteristics can differ in significant ways.

In conclusion, we found that emotional stability is associated with loneliness across two generations of older people. Social circumstances appear to be more related to loneliness in the older of the two generations (70–79 years old), and extraversion appears to be more related to loneliness in the younger of the two generations (55–69 years old). Future work ought to further investigate generational differences in the sources of loneliness, with the aim of determining whether these differences come about as the result of ageing, or if there are fundamental differences in how generations use their social networks to connect with other people.

## Supplementary material

For supplementary material accompanying this paper visit https://doi.org/10.1017/S0033291719003933. click here to view supplementary material

## Open practices statement

The analyses were planned in exploratory-confirmatory pairs rather than being pre-registered. Source data are not publicly available, but all can be requested from the individual study organizers. Resultant data are available as supplementary data with the journal, hosting code as supplementary material is not recommended as best practice.
