# Supplementary material for: Generational differences in loneliness and its psychological and sociodemographic predictors: an exploratory and confirmatory machine learning study
Source: Psychol Med. 2020 Mar 9;51(6):991–1000. doi: 10.1017/S0033291719003933 (PMC8161432; doi:10.1017/S0033291719003933)
Supplement: Supplementary file 1 [file S0033291719003933sup001.docx]

**Supplementary Methods, Tables, and Figures**

Cohorts selection.

*Thirty-Six Day Sample*

On June 4, 1947, almost all children born in 1936 and attending school in Scotland completed the Scottish Mental Survey of 1947 (SMS1947), organized by the Scottish Council for Research in Education. As part of a sub-study, a representative subsample was selected according to their dates of birth being on one of the first 3 days of each month (i.e., 36 days throughout the year). This was known as the Thirty-Six Day Sample (36DS; N = 7277). This sample was initially followed-up in the same year (1947) with a sociological survey.

In 2012 and 2013, members of the 36DS were traced through the UK National Health Service Central Register (Brett and Deary, 2014). Those who were alive and living in Scotland, England, or Wales (N= 2977) were invited to participate in a follow-up, at around age 78 years. 722 individuals completed a detailed questionnaire and 365 individuals later completed a telephone interview (Brett and Deary, 2014, Deary and Brett, 2015). 36DS was used for exploratory analyses in the older generation.

*Lothian Birth Cohort of 1936*

The Lothian Birth Cohort of 1936 (LBC1936) also has its origins in the SMS1947. Between 2004 and 2007, 1091 participants of the SMS1947 were recruited to Wave 1 of the LBC1936 study, at a mean age of 70 years (Taylor et al., 2018). Large amounts of other cognitive, psychosocial, lifestyle, and other data were collected at wave 1 and in three subsequent waves, at ages 73, 76, and 79. 76 members of the LBC1936 were also part of the 36DS; these individuals were removed from the LBC1936 sample and thus only used in exploratory analyses. The remaining LBC1936 sample was used for confirmatory analyses in the older generation.

Younger samples.

*Healthy Ageing in Scotland*

Healthy Ageing in Scotland (HAGIS) is a proposed longitudinal study of older individuals living in Scotland. Participants were identified by household; everyone in the household was invited who met one of the following two conditions: 1) adults who were at least 50 years of age at the time of data collection, 2) partners of adults aged 50 years or older who were themselves 45 years or older. HAGIS currently consists of an initial pilot sample of 1000 participants, who were assessed via a face-to-face computer assisted personal interview and a shorter questionnaire that participants could complete themselves (Douglas et al., 2018). HAGIS’s survey data were designed to be similar to and harmonizable with other global ageing studies, such as the Health and Retirement Study in the USA. HAGIS was used for exploratory analyses in the younger generation. Despite including individuals older than 70, to ensure that there was no age overlap between the younger and older samples, after variables were selected and processed, individuals older than 69 years were removed from our analytic HAGIS sample, leaving 612 participants.

*English Longitudinal Study of Ageing*

The English Longitudinal Study of Ageing (ELSA) is a panel study of a representative cohort of English men and women (Steptoe et al., 2012). Started in 2002, the initial sample was invited from participants in the 1998, 1999, and 2000 Health Survey for England if they would be 50 years or older by the start of the first wave of data collection. Subsequent waves have been collected approximately every 2 years. We analyzed wave 2 of ELSA, the first with a measure of loneliness, which included 9432 participants. These data were collected in 2004 and 2005, when participants were 59 years old on average. Computer-assisted personal interviews and self-completion questionnaires were used to collect psychosocial data, and ELSA was also designed to be comparable to and harmonizable with other global ageing studies, such as the Health and Retirement Study. ELSA was used for confirmatory analyses in the younger generation. As with HAGIS, for analysis, individuals older than 69 at the time of wave 2 data collection were removed from our analytic ELSA sample, leaving 6106 participants.

*Personality scoring.*

36DS used the 20-item mini-IPIP, scored as shown below:

| Table S1. 36DS, 20-item IPIP scoring. |  |  |
| --- | --- | --- |
|  |  |  |
| Item | Dimension | Sign |
| Am the life of the party | Extraversion | + |
| Don’t talk a lot | Extraversion | — |
| Talk to a lot of different people at parties | Extraversion | + |
| Keep in the background | Extraversion | — |
| Sympathize with others' feelings | Agreeableness | + |
| Am not interested in other people's problems | Agreeableness | — |
| Feel others' emotions | Agreeableness | + |
| Am not really interested in others | Agreeableness | — |
| Get chores done right away | Conscientiousness | + |
| Often forget to put things back in their proper place | Conscientiousness | — |
| Like order | Conscientiousness | + |
| Make a mess of things | Conscientiousness | — |
| Have frequent mood swings | Emotional stability | + |
| Am relaxed most of the time | Emotional stability | — |
| Get upset easily | Emotional stability | + |
| Seldom feel blue | Emotional stability | — |
| Have a vivid imagination | Intellect | + |
| Am not interested in abstract ideas | Intellect | — |
| Have difficulty understanding abstract ideas | Intellect | — |
| Do not have a good imagination | Intellect | — |
|  |  |  |

LBC1936 and HAGIS used the 50-item IPIP, scored as shown below:

| Table S2. LBC1936 and HAGIS, 50-item IPIP scoring. |  |  |
| --- | --- | --- |
|  |  |  |
| Item | Dimension | Sign |
| Am the life of the party | Extraversion | + |
| Don’t talk a lot | Extraversion | — |
| Talk to a lot of different people at parties | Extraversion | + |
| Keep in the background | Extraversion | — |
| Feel comfortable around people | Extraversion | + |
| Start conversations | Extraversion | + |
| Have little to say | Extraversion | — |
| Don't like to draw attention to myself | Extraversion | — |
| Don't mind being the center of attention | Extraversion | — |
| Am quiet around strangers | Extraversion | + |
| Sympathize with others' feelings | Agreeableness | + |
| Am not interested in other people's problems | Agreeableness | — |
| Feel others' emotions | Agreeableness | + |
| Am not really interested in others | Agreeableness | — |
| Feel little concern for others | Agreeableness | — |
| Am interested in people | Agreeableness | + |
| Insult people | Agreeableness | — |
| Have a soft heart | Agreeableness | + |
| Take time out for others | Agreeableness | + |
| Make people feel at ease | Agreeableness | + |
| Get chores done right away | Conscientiousness | + |
| Often forget to put things back in their proper place | Conscientiousness | — |
| Like order | Conscientiousness | + |
| Make a mess of things | Conscientiousness | — |
| Am always prepared | Conscientiousness | + |
| Leave my belongings around | Conscientiousness | — |
| Pay attention to details | Conscientiousness | + |
| Shirk my duties | Conscientiousness | — |
| Follow a schedule | Conscientiousness | + |
| Am exacting in my work | Conscientiousness | + |
| Have frequent mood swings | Emotional stability | — |
| Am relaxed most of the time | Emotional stability | + |
| Get upset easily | Emotional stability | — |
| Seldom feel blue | Emotional stability | + |
| Get stressed out easily | Emotional stability | — |
| Worry about things | Emotional stability | — |
| Am easily disturbed | Emotional stability | — |
| Change my mood a lot | Emotional stability | — |
| Get irritated easily | Emotional stability | — |
| Often feel blue | Emotional stability | — |
| Have a vivid imagination | Intellect | + |
| Am not interested in abstract ideas | Intellect | — |
| Have difficulty understanding abstract ideas | Intellect | — |
| Do not have a good imagination | Intellect | — |
| Have a rich vocabulary | Intellect | + |
| Spend time reflecting on things | Intellect | + |
| Have excellent ideas | Intellect | + |
| Am quick to understand things | Intellect | + |
| Use difficult words | Intellect | + |
| Am full of ideas | Intellect | + |
|  |  |  |

ELSA used a 26-item version of the Midlife Development Inventory (MIDI), scored as shown below:

| Table S3. ELSA, 26-item MIDI scoring. |  |  |
| --- | --- | --- |
|  |  |  |
| Adjective | Dimension | Sign |
| Outgoing | Extraversion | + |
| Friendly | Extraversion | + |
| Active | Extraversion | + |
| Talkative | Extraversion | + |
| Lively | Extraversion | + |
| Warm | Agreeableness | + |
| Helpful | Agreeableness | + |
| Soft-hearted | Agreeableness | + |
| Sympathetic | Agreeableness | + |
| Caring | Agreeableness | + |
| Organized | Conscientiousness | + |
| Responsible | Conscientiousness | + |
| Thorough | Conscientiousness | + |
| Hardworking | Conscientiousness | + |
| Careless | Conscientiousness | — |
| Moody | Emotional stability | — |
| Worrying | Emotional stability | — |
| Nervous | Emotional stability | — |
| Calm | Emotional stability | + |
| Creative | Intellect | + |
| Imaginative | Intellect | + |
| Intelligent | Intellect | + |
| Curious | Intellect | + |
| Sophisticated | Intellect | + |
| Adventurous | Intellect | + |
|  |  |  |

Items were not evenly distributed across all personality domains, so each personality score used in the statistical analyses is the mean score of all answered items for each domain, so that like the IPIP measures, each dimension can be more easily compared with others.

*Cognitive testing.*

Independent principal components analyses (PCA) were conducted within each sample, using the cognitive tests available. The variables included in and the results of each PCA are described below for each sample.

36DS included the National Adult Reading Test (NART), a test of semantic fluency (number of animals an individual could name in 1 minute), a test of delayed word recall, symbol-digit modalities test, and Raven’s standard progressive matrices.

| Table S4. Principal components analysis of 36DS cognitive tests. | | | |
| --- | --- | --- | --- |
|  |  |  |  |
| Test | First component | Communality | Uniqueness |
| NART | 0.61 | 0.37 | 0.63 |
| Semantic fluency | 0.49 | 0.24 | 0.76 |
| Word recall | 0.79 | 0.62 | 0.38 |
| Delayed recall | 0.71 | 0.50 | 0.50 |
| Symbol-digit modalities | 0.76 | 0.57 | 0.43 |
| Progressive matrices | 0.65 | 0.42 | 0.58 |
| SS loadings | 2.72 |  |  |
| Variance explained | 45% |  |  |

LBC1936 included tests of matrix reasoning and block design from the Wechsler Adult Intelligence Scale (WAIS), and spatial span forward and backward from the Wechsler Memory Scale (WMS). Speed tests included symbol search and digit symbol substitution from the WAIS, plus four-choice reaction time and inspection time. Memory tests included verbal paired associates and logical memory from the WMS, and the letter–number sequencing and digit span backward subtests of the WAIS. Finally, vocabulary / fluency was measured through the NART, Wechsler Test of Adult Reading (WTAR) and a phonemic verbal fluency test.

| Table S5. Principal components analysis of HAGIS cognitive tests. | | | |
| --- | --- | --- | --- |
|  |  |  |  |
| Test | First component | Communality | Uniqueness |
| NART | 0.73 | 0.53 | 0.47 |
| WTAR | 0.73 | 0.53 | 0.47 |
| Verbal fluency | 0.56 | 0.32 | 0.68 |
| Letter-number sequencing | 0.71 | 0.51 | 0.49 |
| Progressive matrices | 0.68 | 0.47 | 0.53 |
| Block design | 0.66 | 0.43 | 0.57 |
| Spatial span forwards | 0.42 | 0.18 | 0.82 |
| Spatial span backwards | 0.54 | 0.30 | 0.70 |
| Symbol search | 0.72 | 0.53 | 0.47 |
| Digit-symbol coding | 0.67 | 0.45 | 0.55 |
| Inspection time | 0.40 | 0.16 | 0.84 |
| 4-choice reaction time | -0.56 | 0.32 | 0.68 |
| Logical memory | 0.57 | 0.33 | 0.67 |
| Verbal paired associates | 0.48 | 0.23 | 0.77 |
| Digit span backwards | 0.61 | 0.38 | 0.62 |
| SS loadings | 5.65 |  |  |
| Variance explained | 38% |  |  |
|  |  |  |  |

HAGIS included word recall and delayed recall tests, an animal recall fluency test, letter digit substitution, a vocabulary test, and progressive matrices.

| Table S6. Principal components analysis of HAGIS cognitive tests. | | | |
| --- | --- | --- | --- |
|  |  |  |  |
| Test | First component | Communality | Uniqueness |
| Vocabulary | 0.52 | 0.27 | 0.73 |
| Animal recall fluency | 0.71 | 0.50 | 0.50 |
| Word recall | 0.76 | 0.58 | 0.42 |
| Delayed recall | 0.70 | 0.50 | 0.50 |
| Letter-digit substitution | 0.66 | 0.44 | 0.56 |
| Progressive matrices | 0.60 | 0.36 | 0.64 |
| SS loadings | 2.65 |  |  |
| Variance explained | 44% |  |  |

ELSA included word recall and delayed recall tests, an animal recall fluency test, and a letter search and cancellation test.

| Table S7. Principal components analysis of ELSA cognitive tests. | | |  |
| --- | --- | --- | --- |
|  |  |  |  |
| Test | First component | Communality | Uniqueness |
| Animal recall fluency | 0.72 | 0.51 | 0.49 |
| Word recall | 0.78 | 0.61 | 0.39 |
| Delayed recall | 0.74 | 0.55 | 0.45 |
| Letter cancellation (correct) | 0.82 | 0.68 | 0.32 |
| Letter cancellation (missed) | 0.33 | 0.11 | 0.89 |
| Letter cancellation (total searched) | 0.81 | 0.66 | 0.34 |
| SS loadings | 3.12 |  |  |
| Variance explained | 52% |  |  |
|  |  |  |  |

*Subjective health.*

In 36DS, HAGIS, and ELSA, the 5 point rating scale included, ‘poor’, ‘fair’, ‘good’, ‘very good’, and ‘excellent’, in that order. The main difference between these ratings and LBC1936’s WHO Quality of Life question was in the labeling of the ratings, which included ‘Very satisfied’, ‘Satisfied’, ‘Neither satisfied nor dissatisfied’, ‘Dissatisfied’, and ‘Very dissatisfied’. All subjective health variables were numericized and reoriented such that the worst health was represented by 1 and the best health represented by 5.

*Social class.*

The Standard Occupational Classification (SOC) 2000 categorizes different UK occupations, and ordinal social classes can then be determined. Social class was categorized into six groups: I (professional), II (intermediate), III NM (skilled nonmanual), III M (skilled manual), IV (semi-skilled), and V (unskilled).

Scottish Index of Multiple Deprivation (SIMD) is a weighted aggregation of small-area-based deprivation measures across seven domains: current income, employment, health, education, skills, housing, geographic access, and crime. SIMD is calculated for 6,976 small geographic areas in Scotland, each of which is ranked, and the ranks were linked to each HAGIS participant.

In order to maintain data comparability between exploratory and confirmatory models, ELSA’s social class variable was scaled and centered to match the mean and standard deviation of HAGIS’ SIMD variable; the distribution of ELSA’s social class variable was otherwise unchanged.

*Code.*

Analytic code is available for the exploratory and confirmatory analyses and can be found on GitHub.

**Figure S1. Whisker plot illustrating the interaction between sex and living alone in the older generation on loneliness scores.** 36DS = Thirty-six Day Sample; LBC1936 = Lothian Birth Cohort of 1936.

**
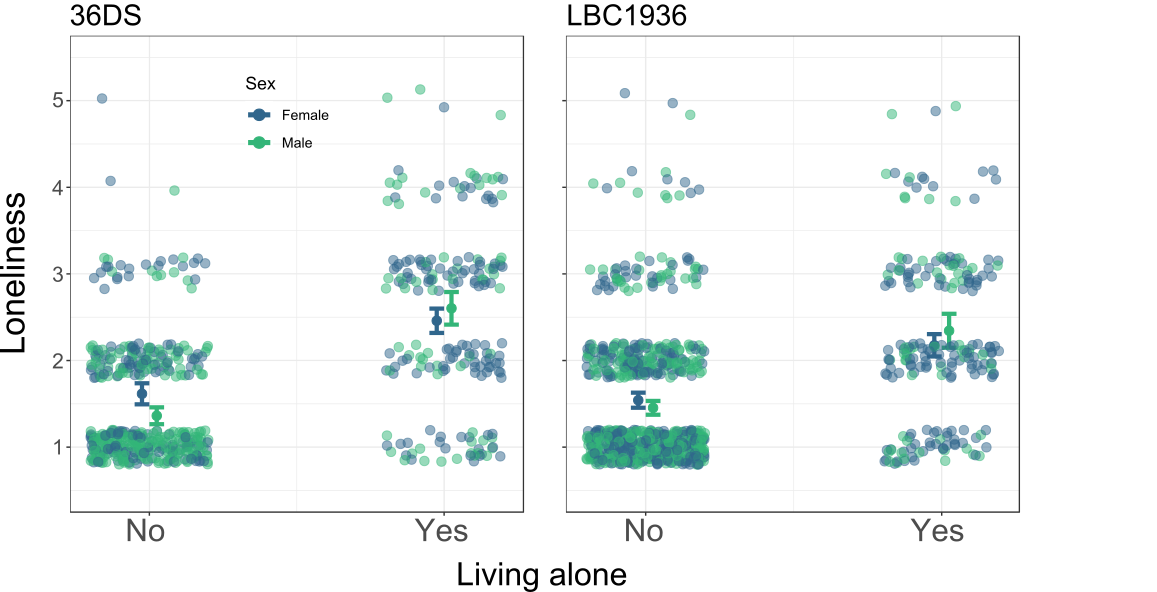
**
